# Significant Increase in Salivary Substance P Level after a Single Oral Dose of Cevimeline in Humans

**DOI:** 10.1155/2013/284765

**Published:** 2013-03-24

**Authors:** Yosuke Suzuki, Hiroki Itoh, Kohei Amada, Ryota Yamamura, Yuhki Sato, Masaharu Takeyama

**Affiliations:** Department of Clinical Pharmacy, Oita University Hospital, Hasama-machi, Oita 879-5593, Japan

## Abstract

Cevimeline is a novel muscarinic acetylcholine receptor agonist currently being developed as a therapeutic agent for xerostomia. We examined the effects of cevimeline on salivary and plasma levels of substance-P- (SP-), calcitonin-gene-related-peptide- (CGRP-), and vasoactive-intestinal-polypeptide- (VIP-) like immunoreactive substances (ISs) in humans. An open-labeled crossover study was conducted on seven healthy volunteers. Saliva volume was measured, and saliva and venous blood samples were collected before and 30–240 min after a single oral dose of cevimeline or placebo. Salivary and plasma levels of SP-, CGRP-, and VIP-IS were measured using a highly sensitive enzyme immunoassay. A single oral dose of cevimeline resulted in significant increases in salivary but not plasma SP-IS level compared to placebo. Cevimeline administration did not alter the salivary or plasma levels of CGRP-IS or VIP-IS compared to placebo. Significant increases in salivary volume were observed after cevimeline administration compared to placebo. A significant correlation was observed between the total release of SP-IS and that of salivary volume. These findings suggest an association of SP with the enhancement of salivary secretion by cevimeline.

## 1. Introduction

**Figure 1:**
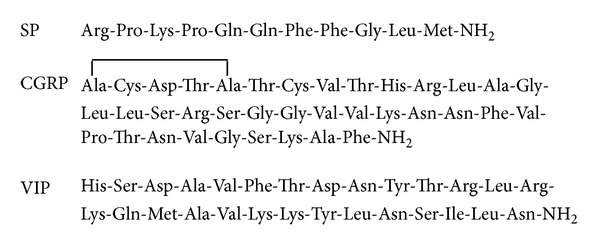
Structures of substance P (SP), calcitonin gene-related peptide (CGRP), and vasoactive intestinal polypeptide (VIP).

The functions of the salivary glands are controlled by the autonomic nervous system and influenced by the sensory nervous system. When parasympathetic impulses dominate, salivary flow is greatly enhanced and the saliva has a low protein content. Studies of animal and human innervation have revealed that parasympathetic nerve fibers are present around acinar cells, ducts, and blood vessels in the major salivary glands [1]. A research has also shown that beside the classic transmitters noradrenaline and acetylcholine, neuropeptides such as substance P (SP), calcitonin gene-related peptide (CGRP), and vasoactive intestinal polypeptide (VIP) (Figure 1) are present in the nerve fibers of the autonomic nervous system as well as in the auriculotemporal nerve, facial nerve, and cervical dorsal root fibers [2]. These neuropeptides are known to cause salivation in rats [2–7]. In recent years, the mechanisms of actions of drugs that used to treat xerostomia have been elucidated pharmacologically from the viewpoint of salivary neuropeptide levels. Anethole trithione and pilocarpine have been shown to elevate SP and CGRP levels in human saliva [8–11].

**Figure 2:**
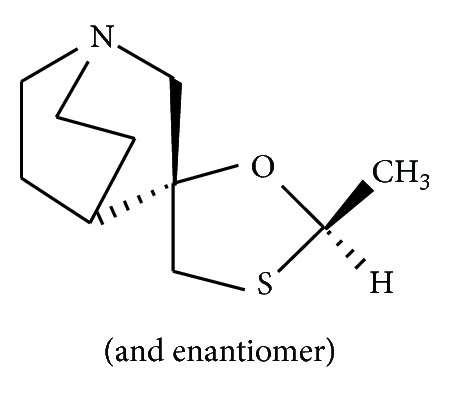
Structure of cevimeline.

Cevimeline hydrochloride hydrate (cevimeline) (Figure 2) is a novel muscarinic acetylcholine receptor agonist currently being developed as a therapeutic agent for Sjögren's syndrome. Sjögren's syndrome is a serious and chronic autoimmune disorder characterized by inflammation in the exocrine glands such as the salivary and lacrimal glands [12], leading to xerostomia (dry mouth) and xerophthalmia (dry eyes). Cevimeline acts as a stimulator of the M3 acetylcholine receptor expressed on salivary glands and has been shown to increase saliva secretion in patients with Sjögren's syndrome [13]. Although cevimeline is useful for the treatment of dry mouth, it only enhances saliva production in 60% of the patients [14], and the mechanism of the drug response is still unknown. It is possible that individual variability of neuropeptide nerve stimulation in response to cevimeline may be involved in the variable drug response to cevimeline. However, the effects of cevimeline in stimulating neuropeptide nerves have not been demonstrated.

The objective of the present study is to examine the effects of cevimeline on saliva and plasma levels of SP-, CGRP-, and VIP-like immunoreactive substances (ISs) in humans, as markers of nerve stimulation of these neuropeptides.

## 2. Materials and Methods

Cevimeline hydrochloride hydrate (Saligren Capsule 30 mg) was purchased from Nippon Kayaku Co. Ltd. (Tokyo, Japan). Lactose (Merck Hoei Co. Ltd., Osaka, Japan) was used as placebo. Synthetic human SP, CGRP and its fragment (8–37), and VIP were purchased from Peptide Institute, Inc. (Osaka, Japan). VIP fragment (11–28) was supplied by Professor Yajima (Kyoto University, Kyoto, Japan). Substance P antiserum (Y150) was purchased from Yanaihara Institute (Shizuoka, Japan), CGRP antiserum (14160) from Peptide Institute, Inc., and VIP antiserum (T-4116) from Peninsula Laboratories (California, USA). All other reagents were analytical reagent grade from commercial sources.

Seven healthy nonsmoking male volunteers aged 24–31 (median 27) years and weighing 56–70 (median 64) kg participated in this study. All subjects had no history of xerostomia, and their baseline fasting salivary and plasma levels of SP-, CGRP-, and VIP-IS were within the normal ranges for healthy subjects reported previously [8–11, 15, 16]. The study was approved by the Ethics Committee of Oita Medical University. Each subject gave informed consent after receiving explanation on the scientific purpose of the study. No subject received any medication during one month before the study. The subjects fasted for at least 2 hours before the study was commenced and during the experiments.

We performed an open-labeled, crossover study between May and October 2010. In each subject, cevimeline and placebo were studied in random order, in a crossover manner with an interval of one month between the two studies. On the day of study, all subjects finished lunch (standardized lunch of less than 800 kcal) before 12:00. Each study was conducted from 14:00 to 18:00 in a room with temperature controlled at 25°C, during which the subjects maintained a resting and relaxed state. A single dose of cevimeline 30 mg (cevimeline group) or placebo (placebo group) was administered orally with 100 mL water. At scheduled times after the test drug was administered, saliva production was measured, and saliva samples were collected for assaying salivary neuropeptide levels, while blood samples were collected for measuring plasma neuropeptide levels. The dose of cevimeline in this study was the normal daily dose used in clinical therapy. Saliva and venous blood samples were collected before and at 30, 60, 90, 120, 180, and 240 min after administration of cevimeline or placebo.

The volume of saliva produced in 5 min was measured by the Saxon test, an oral equivalent of the Schirmer test [17]. Two sterile absorbent cotton balls (no. 14, Kawamoto Houtai Zairyou, Osaka, Japan) and a polyethylene pouch were weighed. After swallowing to remove any existing oral fluid, saliva was collected by placing the two cotton balls onto the vestibule of the mouth for exactly 5 min. The subjects then expectorated the moist absorbent cotton balls into the polyethylene pouch. The weight of saliva was determined by subtracting the original weight of the pouch and cotton balls from the weight obtained after the cotton balls were placed in the mouth. The weight of the liquid was taken to be the salivary volume (mL) produced in 5 minutes.

Unstimulated whole saliva specimens were collected by the spitting method according to Navazesh and Christensen [18]. The subjects rinsed their mouth thoroughly with deionized water and rested for a few minutes before saliva collection began. After one minute practice collection, which was discarded, subsequently 3 mL of saliva was collected into a test tube containing 500 kallikrein inhibitor units/mL of aprotinin and 1.2 mg/mL of EDTA. Blood samples were collected into chilled tubes containing 500 kallikrein inhibitor units/mL of aprotinin and 1.2 mg/mL of EDTA.

The saliva samples were diluted 1 : 1 with 4% acetate buffer (pH 4.0), centrifuged at 3500 rpm for 5 min at 4°C, and then the supernatant was diluted 2 : 3 with 4% acetate buffer (pH 4.0) and loaded onto C18 reverse-phase cartridges (Sep-Pak C18; Millipore Corp., Milford, MA, USA). Blood samples were centrifuged, and the plasma samples were diluted 1 : 4 with 4% acetate buffer (pH 4.0) and loaded onto C18 reverse-phase cartridges. After washing with 4% acetate buffer, neuropeptides in the columns were eluted with 70% acetonitrile in 0.5% acetate buffer (pH 4.0). Eluates were concentrated by spin-vacuum evaporation, lyophilized, and stored at −40°C until use. The recovery of SP-, CGRP-, and VIP-IS in saliva and plasma was greater than 90% using this extraction procedure [19–21].

Neuropeptide levels in saliva and plasma were measured using highly sensitive enzyme immunoassays for SP [19], CGRP [20], and VIP [21] as described previously. The assays were performed by a delayed addition method. An immunoplate (Nunc-Immuno Module Maxisorp F8, InterMed, Denmark) coated with anti-rabbit IgG (55641, ICN Pharmaceuticals, Inc., Ohio, USA) was used to separate bound and free antigens. Human SP, CGRP fragment (8–37), or VIP fragment (11–28) was conjugated with *β*-D-galactosidase by *N*-(*ε*-maleimido-caproyloxy)-succinimide according to the methods of Kitagawa et al. [22]. The enzyme immunoassays were specific and highly sensitive, with detection limits of 0.08, 0.40, and 1.00 fmol/well for SP-, CGRP-, and VIP-IS, respectively.

All values are expressed as means ± standard deviation (SD). Total release of each neuropeptide or saliva was calculated as the area under the level—or volume—time curve (AUC_0–240_) using the trapezoidal method. Differences in neuropeptide-IS level, salivary volume, and their AUC_0–240_ between the cevimeline and placebo groups were analyzed by paired *t*-test or Mann-Whitney *U* test. The relationship between AUC_0–240_ of neuropeptide-IS level and AUC_0–240_ of salivary volume was analyzed by Pearson's product-moment correlation coefficient. A *P* value less than 0.05 was considered statistically significant. Statistical analyses were performed using the SPSS software package (version 17.0; SPSS Inc., IL, USA).

## 3. Results

**Figure 3:**
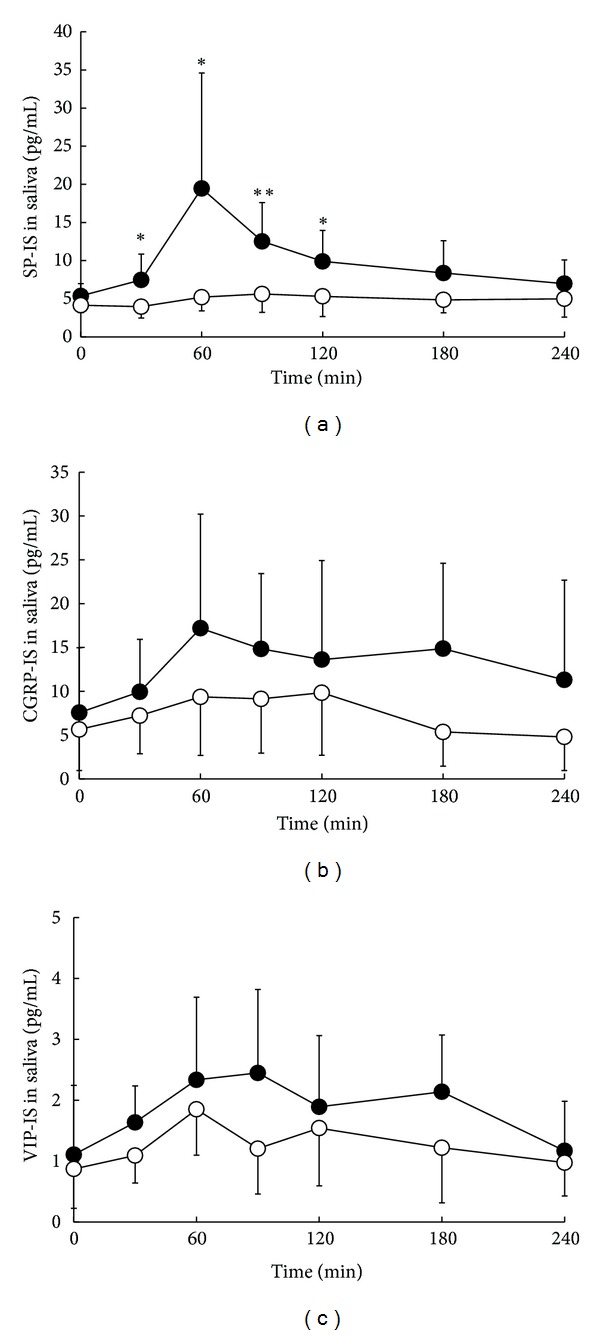
Effects of a single oral dose of cevimeline (•) or placebo (°) on salivary levels of substance P (SP) (a), calcitonin gene-related peptide (CGRP) (b), and vasoactive intestinal polypeptide (VIP) immunoreactive substance (c). Values are means ± SD, *n* = 7. *P < 0.05, **P < 0.01, versus placebo at the same time point.

The salivary SP-IS level-time profile and total release of SP-IS (AUC_0–240_) after a single oral dose of cevimeline or placebo are shown in Figure 3(a) and Table 1. Oral administration of cevimeline resulted in significant increases in salivary SP-IS level at 30, 60, 90, and 120 min (7.5 ± 3.4, 19.1 ± 15.1, 12.5 ± 5.1, and 9.9 ± 4.1 pg/mL, resp.) compared with the corresponding levels after placebo administration (4.0 ± 1.5, 5.2 ± 1.8, 5.6 ± 2.4, and 5.3 ± 2.7 pg/mL). Furthermore, AUC_0–240_ was significantly higher after cevimeline administration (2420.9 ± 744.6 pg·min/mL) compared with placebo (1185.8 ± 398.6 pg·min/mL). On the other hand, no significant changes in salivary CGRP- and VIP-IS levels and AUC_0–240_ were observed after the administration of cevimeline (Figures 3(b) and 3(c) and Table 1) compared with placebo.

**Figure 4:**
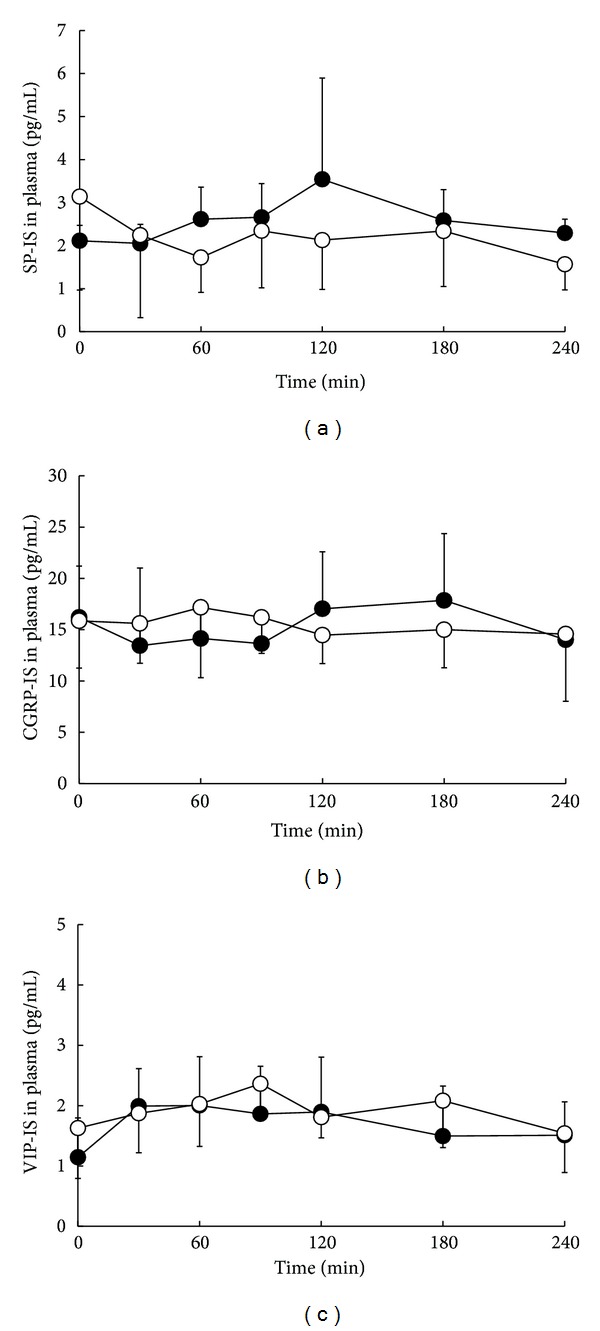
Effects of a single oral dose of cevimeline (•) or placebo (°) on plasma levels of substance P (SP) (a), calcitonin gene-related peptide (CGRP) (b), and vasoactive intestinal polypeptide (VIP) immunoreactive substance (c). Values are means ± SD, *n* = 7.

The plasma SP-, CGRP-, and VIP-IS level-time profiles and total releases of SP-, CGRP-, and VIP-IS (AUC_0–240_) after a single oral dose of cevimeline or placebo are shown in Figure 4 and Table 2. Cevimeline administration did not alter the plasma levels or AUC_0–240_ of SP-, CGRP-, or VIP-IS compared with placebo.

**Figure 5:**
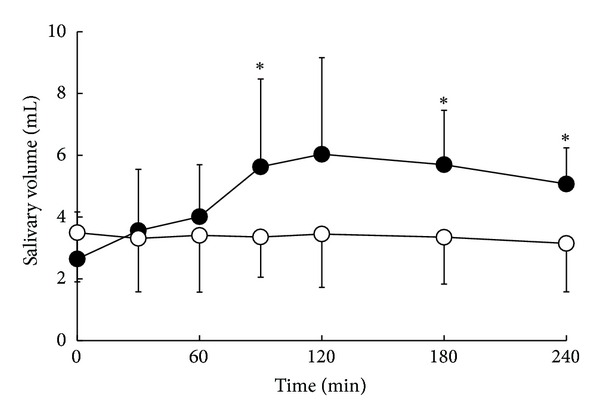
Effects of a single oral dose of cevimeline (•) or placebo (°) on salivary volume. Values are means ± SD, *n* = 7. *P < 0.05 versus placebo at the same time point.

The changes in salivary volume and total release of saliva (AUC_0–240_) after cevimeline or placebo administration are shown in Figure 5 and Table 3. Cevimeline administration resulted in significant increases in salivary volume at 90, 180, and 240 min (5.6 ± 2.8, 5.7 ± 1.8, and 5.1 ± 1.2 mL, resp.) compared with the corresponding levels after placebo administration (3.4 ± 1.3, 3.4 ± 1.5, and 3.2 ± 1.6 mL). The AUC_0–240_ was also significantly higher after cevimeline administration (1200.8 ± 403.4 mL·min) compared with placebo (804.9 ± 369.8 mL·min).

**Figure 6:**
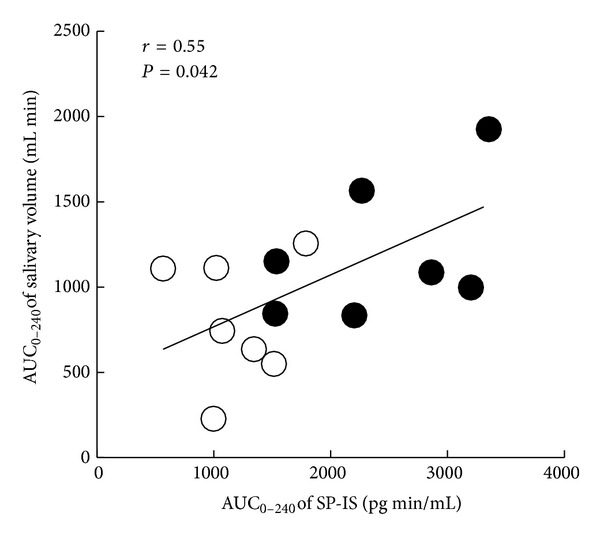
Relationship between the area under the time curve (AUC_0–240_) of substance P (SP)-immunoreactive substance (IS) level and AUC_0–240_ of salivary volume after a single oral dose of cevimeline (•) or placebo (°).

The relationship between AUC_0–240_ of SP-IS level and salivary volume after administration of cevimeline or placebo is shown in Figure 6. A significant correlation was observed between AUC_0–240_ of SP-IS level and AUC_0–240_ salivary volume (*r* = 0.55, *P* = 0.042).

## 4. Discussion

In this study, we investigated the effects of cevimeline on saliva and plasma levels of SP-, CGRP-, and VIP-IS in healthy subjects. Past studies have established that salivary and plasma levels of SP-, CGRP-, and VIP-IS vary within 30 min after a meal and then maintain constant from 1 hour after a meal [8, 9, 19–21]. Furthermore, it is known that the absorption of cevimeline is little affected by a meal. These data support our study design, and the present study appropriately evaluates the effects of cevimeline on neuropeptide levels and saliva production without being affected by a meal.

Neuropeptides such as SP, CGRP, and VIP are important stimulators of salivation. SP is mainly localized in submandibular and parotid glands and increases blood flow via its vasodilatory effect in salivary glands, stimulates the production of saliva and amylase, and influences ionic flow in rats [23, 24]. Previous report indicates that CGRP also enhances the release of saliva and amylase in rats [3, 6], and VIP induces alterations in salivary fluid and protein secretion [4, 25]. In the present study, a single oral dose of cevimeline resulted in significant increases in salivary SP-IS level at 30, 60, 90, and 120 min and in the AUC_0–240_ of SP-IS compared with placebo administration, whereas cevimeline did not alter the plasma levels or AUC_0–240_ of SP-IS. Anethole trithione and pilocarpine have also been reported to increase SP-IS in saliva but not in plasma [8–11]. These results indicate a close association of SP with the enhancement of salivary secretion by cevimeline, in the same manner as anethole trithione and pilocarpine. In addition, these findings suggest that cevimeline may mainly promote salivary secretion from submandibular and parotid glands by increasing SP. On the other hand, no significant changes in salivary and plasma levels and AUC_0–240_ of CGRP- and VIP-IS were observed after the administration of cevimeline. These findings suggest that pathways via CGRP and VIP nerves may not be involved in the stimulatory effect on salivation by cevimeline. On the other hand, anethole trithione and pilocarpine increase not only SP but also CGRP levels in human saliva [8–11]. Cevimeline acts as a selective stimulator of the M3 acetylcholine receptor expressed on salivary glands [13], and this selectivity may reflect the specificity of the cevimeline action on SP nerves in salivary glands.

Oral cevimeline administration resulted in significant increases in salivary volumes at 90, 180, and 240 min and in the AUC_0–240_ compared with placebo administration. Furthermore, a significant correlation was observed between AUC_0–240_ of SP-IS level and AUC_0–240_ of salivary volume, suggesting the possible involvement of SP in the cevimeline-enhanced saliva secretory activity. A lag time was observed between elevation of salivary SP level and increase in salivary volume, suggesting that SP secreted from the SP nerves stimulated by cevimeline may initially increase blood flow and cause vasodilatation in salivary glands, followed by a gradual increase in salivary production. However, some reports have suggested that human salivary glands are thought to lack an SP innervation of the acinar cells, and in vitro pieces of human submandibular glands do not respond with fluid secretion to the administration of SP, as judged by the release of potassium [26, 27]. Furthermore, other neuropeptides not tested in this study may also be involved in the mechanism of enhancement of salivary secretion by cevimeline. Therefore, this notion requires verification by further studies.

Cevimeline is known to enhance saliva production in only 60% of the treated patients [14], and the mechanism of drug response remains unknown. The present study shows a possibility that individual variability of SP nerve stimulation in response to cevimeline may account for the variable drug response to cevimeline, although it is uncertain whether this trend in healthy volunteers is also observed in patients. Therefore, further studies are required to investigate the effects of cevimeline in patients with conditions such as xerostomia.

## 5. Conclusions

This study demonstrated the effects of cevimeline on salivary and plasma levels of neuropeptides in humans. A single oral dose of cevimeline resulted in a significant increase in salivary but not plasma SP-IS level, and a significant correlation was observed between the total release of salivary SP-IS and of salivary volume. These findings suggest a close association of SP with the enhancement of salivary secretion by cevimeline. A large-scale controlled study evaluating multiple dosing conditions of cevimeline would help to better understand the effects of cevimeline.

## Figures and Tables

**Table 1:** Total amounts of substance-P- (SP-), calcitonin-gene-related-peptide- (CGRP-), and vasoactive-intestinal-polypeptide-(VIP-) like immunoreactive substance in saliva released after administration ofcevimeline or placebo to 7 healthy volunteers.

Drugs	AUC_0–240_ in saliva (pg min/mL)		
	SP	CGRP	VIP
Cevimeline	2420.9 ± 744.6**	3014.4 ± 2009.3	389.8 ± 120.4
Placebo	1185.8 ± 398.6	1644.1 ± 1094.7	288.3 ± 125.7

Data are expressed as mean ± SD, **P < 0.01 versus placebo.

**Table 2:** Total amounts of substance-P- (SP-), calcitonin-gene-related-peptide- (CGRP-), and vasoactive-intestinal-polypeptide-(VIP-) like immunoreactive substance in plasma released after administration of cevimeline or placebo to 7 healthy volunteers.

Drugs	AUC_0–240_ in plasma (pg min/mL)		
	SP	CGRP	VIP
Cevimeline	453.4 ± 343.6	2518.0 ± 1841.0	295.1 ± 210.4
Placebo	445.3 ± 316.2	2640.4 ± 1936.1	398.4 ± 189.3

Data are expressed as mean ± SD.

**Table 3:** Total amount of saliva released after administration of cevimeline or placebo to 7 healthy volunteers.

Drugs	AUC_0–240_ of salivary volume (mL min)
Cevimeline	1200.8 ± 403.4**
Placebo	804.9 ± 369.8

Data are expressed as mean ± SD, **P < 0.01 versus placebo.
